# Exopthalmia

**Published:** 1856-03

**Authors:** Edwin R. Maxson

**Affiliations:** Geneva, N. Y.


ART. II.—*Exopthalmia.* By Edwin R. Maxson, M. D., of Geneva, N. Y.

Exopthalmia, or protrusion of the eyes, occurring in anæmic females, in connection with enlargement of the thyroid gland, with nervous palpitation, and frequently with amenorrhœa, has become a matter now quite generally understood, since cases have been reported by several eminent physicians in this country and in Europe.

As, however, such cases are quite rare, and as they are liable to be mistaken for enlargement of the eye itself, I will offer a few suggestions in relation to this affection, as it has developed itself in cases falling under my observation.

*Symptoms.* After an anæmic condition has continued for a time, with its attendant symptoms, especially of palpitation, and generally of amenorrhœa, there is discovered an enlargement of the thyroid gland, and soon what appears to be an enlargement of one or both eyes, but which is really a protrusion of the eye, making it appear larger as it comes forward in the orbit.

This prominence of the eye is slight at first, giving the patient a wildish appearance; but it gradually increases till the eyes appear very large, and the appearance of the patient becomes frightful, as the lids fail to cover the eyes even with an effort.

The protrusion varies in some cases within a few hours, a slight cephalic tendency increasing the prominence; while, as the circulation becomes equalized, the eyes assume more nearly their natural position.

This variation I noticed quite often, in one marked case of this affection, which fell under my care a few years since. In this case there was a continued enlargement of the thyroid gland, nervous palpitation, and amenorrhœa.

But it should be remembered that goitre, though a general, is not a universal complication; and the same is also true in relation to the nervous palpitation and amenorrhœa.

*Pathology.* In relation to the pathology of this very singular affection, there may be room for doubt, but a few facts in relation to it appear clear.

It always occurs, so far as I have been able to ascertain, in anæmic patients, and generally in females. It is usually preceded by indigestion, and attended with more or less derangement of the absorbant lymphatic, and glandular system; a condition which evidently favors the enlargement of the thyroid gland, and also a chronic thickening of the tissues in the posterior part of the orbit, which form the cushion of the eye.

The muscles of the eye in an anæmic patient, evidently become weak and more or less relaxed, as does the heart muscle in cases of dilatation of the heart occurring from the same, or a similar condition.

Now, with a weak or watery state of the blood, a relaxed condition of the muscles of the eye, and a deranged condition of the absorbant lymphatic and glandular system, it is easy to see how this variety of exopthalmia may occur. For the anæmic condition favors cephalic congestion, the eye and the orbital tissues being as a consequence more or less congested, and the muscles of the eve are more or less relaxed, the eyes protrude from the orbit, and assume a prominent and enlarged appearance, more especially at first, during slight cephalic congestion. But by degrees a thickening of the tissues in the posterior part of the orbit takes place; the protrusion of the eyes becomes more constant, and gradually increases in the same manner, and from the same condition of the system, which produces the enlargement of the thyroid gland.

Relaxation then of the muscles of the eye, with congestion at first, and then a gradual thickening of the tissues back of the eye in the posterior orbital region, is the immediate condition upon which the protrusion of the eyes depend, in this variety of exopthalmia.

*Treatment.* The indications in the treatment of this affection are plain. A good nourishing diet, taken with regularity; sufficient exercise in the open air; sponging the surface of the body once or twice per week with moderately tepid water containing a little salt, and wearing flannel next the skin, are important indications which should not be neglected.

If the bowels are constipated, a pill of aloes and rhei. $\overline{aa}$ gr. jss. should be given each day after dinner, till they are regulated, and then the iodide of potassium in gr. v. doses, three times per day, should be given, and continued for several weeks; after which the syrup ferri iodid., in gtt. x. doses, should be given, and continued as long as may be required.

GENEVA, N. Y., January 28th, 1856.

